# Albumin-based nanoparticles: a promising strategy to overcome cancer drug resistance

**DOI:** 10.20517/cdr.2020.68

**Published:** 2020-11-03

**Authors:** Islam Hassanin, Ahmed Elzoghby

**Affiliations:** ^1^Department of Biotechnology, Institute of Graduate studies and Research, Alexandria University, Alexandria 21526, Egypt.; ^2^Cancer Nanotechnology Research Laboratory (CNRL), Faculty of Pharmacy, Alexandria University, Alexandria 21521, Egypt.; ^3^Department of Industrial Pharmacy, Faculty of Pharmacy, Alexandria University, Alexandria 21521, Egypt.

**Keywords:** Albumin, active targeting, cancer therapy, drug delivery, multi-drug resistance, nanoparticles, stimuli- response release, nucleic acid therapy

## Abstract

Circumvention of cancer drug resistance is one of the major investigations in nanomedicine. In this regard, nanotechnology-based drug delivery has offered various implications. However, protein-based nanocarriers have been a versatile choice compared to other nanomaterials, provided by their favorable characteristics and safety profiles. Specifically, albumin-based nanoparticles have been demonstrated to be an effective drug delivery system, owing to the inherent targeting modalities of albumin, through gp60- and SPARC-mediated receptor endocytosis. Furthermore, surface functionalization was exploited for active targeting, due to albumin’s abundance of carboxylic and amino groups. Stimuli-responsive drug release has also been pertained to albumin nano-systems. Therefore, albumin-based nanocarriers could potentially overcome cancer drug resistance through bypassing drug efflux, enhancing drug uptake, and improving tumor accumulation. Moreover, albumin nanocarriers improve the stability of various therapeutic cargos, for instance, nucleic acids, which allows their systemic administration. This review highlights the recent applications of albumin nanoparticles to overcome cancer drug resistance, the nano-fabrication techniques, as well as future perspectives and challenges.

## Introduction

Cancer is the uncontrolled proliferation of abnormal cells, which can be treated by various strategies^[1-3]^. Surgery, radiotherapy, hormone therapy, targeted drug therapy, chemotherapy, and immunotherapy are successful therapeutic options for many patients. However, resistance to these treatments may develop, especially for chemotherapy, which may compromise the efficacy of cancer treatment^[4,5]^.

**Figure 1:**
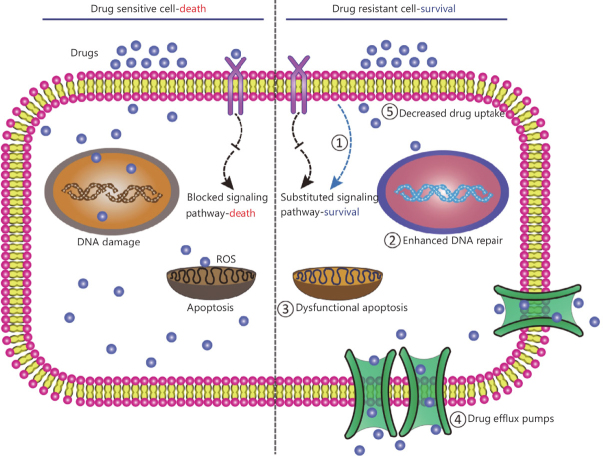
Schematic representation of various cancer drug resistance mechanism^[14]^

Multiple mechanisms participate in the development of cancer resistance [Figure 1]. Intrinsic factors may include: (1) genetic variations, which are comprised of gene mutations, amplifications, deletions, or alterations of miRNA; and (2) epigenetic variations, which are attributed to transcriptomic or proteomic variations. Extrinsic factors are related to pH, hypoxia, and interaction with other tumor cells. Collectively, these factors contribute to the heterogeneity of tumors^[3]^. Moreover, drug inactivation, drug efflux, reduced drug uptake, resistance to apoptosis, enhanced DNA repair mechanisms, and immunosuppression and immune evasion are cancer resistance mechanisms which may negatively influence anti-cancer drug treatment^[6-8]^.

Several strategies have been proposed to overcome acquired cancer resistance mechanisms, including knocking-down of genes responsible for resistance, drug efflux inhibitors, bypassing drug uptake and drug efflux by receptor-mediated endocytosis^[9]^, utilizing stimuli-responsive drug carriers^[10,11]^, or targeting multiple pathways through combination treatments^[12,13]^. These strategies may be implemented via nanotechnology-based delivery systems. Nanocarriers may offer several advantages, such as combined drug use, passive and active targeting, enhanced tumor accumulation, overcoming poor pharmacokinetic profiles of the anticancer agents, improving the stability of the loaded cargo, and tuned drug release profiles^[14-16]^.

Protein nanocarriers, especially albumin-based nanoparticles, offer multiple advantages compared to other nanomaterials. Biocompatibility, biodegradability, less immunogenicity, and lower cytotoxicity are favorable characteristics of protein nanocarriers^[17]^. Moreover, proteins possess an intrinsic ability to target tumor cells, either passively or actively, through overexpressed receptors. Functional groups, necessary for functionalization with targeting materials or active therapeutics, are available for various proteins^[18,19]^.

Albumin-based nanocarriers are a versatile choice, implemented to overcome drug resistance mechanisms for multiple types of tumors. Albumin nanoparticles can be synthesized utilizing various approaches^[20]^, and they offer significant implications including passive and active targeting^[21,22]^, controlled drug release, and, more importantly, bypassing cancer drug resistance mechanisms, such as poor drug uptake or drug efflux mechanisms, as well as inhibition of apoptosis. This review highlights the recent methodologies for the fabrication of albumin-based nanoparticles, application of these nanoparticles in overcoming cancer drug resistance, and future perspectives and challenges.

## Albumin

Human serum albumin (HSA) is a globular plasma protein, which is comprised of 585 amino-acids, and possesses a molecular weight of 66,500 Dalton (Da)^[23,24]^. HSA is composed of three homologous domains, forming a heart-shaped molecule. It is stable against a wide range of factors including temperature, pH (4-9), and organic solvents. Bovine Serum Albumin (BSA) possesses a molecular weight of 69,323 Da. BSA has gained considerable attention in the pharmaceutical industry, owing to low cost, high abundance, and ease of purification. However, compared to HSA, BSA may induce unfavorable immunogenic reactions^[25,26]^.

Albumin is a versatile biomaterial for synthesis of nanoparticles (NPs)^[27]^. The efficiency of the albumin-based delivery resides in its ability to enhance tumor targeting and accumulation. For instance, enhanced tumor accumulation is due to the enhanced uptake passively mediated by the enhanced permeability and retention effect^[28]^. Furthermore, albumin can bind to special receptors overexpressed on cancer cells and enhance nanoparticles binding and internalization. Various tumors overexpress the 60-kDa glycoprotein (gp60) receptor^[29]^, as well as secreted protein acidic and rich in cysteine (SPARC)^[30]^. Albumin can specifically bind to gp60 and SPARC, and, thus, it can actively increase the uptake of the nanoparticles. This particular uptake mechanism allows the albumin-based nanoparticles to bypass the drug efflux mechanisms in tumor cells. It was shown that the binding of paclitaxel, exhibited by nab-paclitaxel, to the endothelium was enhanced by 9.9 folds, and paclitaxel was transported more efficiently by 4.2 folds, compared to Cremophor EL-paclitaxel^[27]^.

Albumin also possesses functional groups, such as amino and carboxylic groups, which can be employed for functionalization of albumin nanoparticles with targeting ligands or active therapeutics^[31]^. Additionally, the stability of albumin nanoparticles allows the systematic delivery of various agents without degradation. For example, albumin nanoparticles successfully delivered siRNA and plasmid-based RNA interference agents^[32,33]^.

## Fabrication techniques of albumin-based nanocarriers employed for overcoming cancer drug resistance

### Desolvation

**Figure 2:**
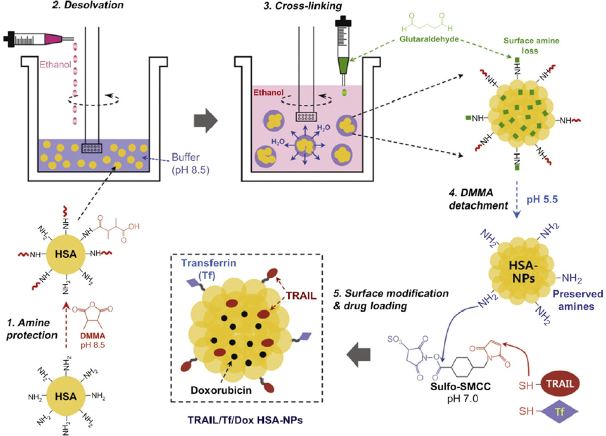
Schematic representation for the preparation of DOX-loaded HSA NPs by desolvation technique, as well as surface functionalization with Transferrin (Tf) and Tumor necrosis factor (TNF)-related apoptosis-inducing ligand (TRAIL)^[37]^. DOX: doxorubicin; HSA: human serum albumin; NPs: nanoparticles

Desolvation is the most common technique used for fabrication of albumin nanoparticles. The process of desolvation is brought by a dehydration process of albumin. This dehydration process is induced when a desolvating agent, for instance, ethanol or acetone, is added to the aqueous solution of albumin. This process induces a conformational change in albumin structure from a stretched to a coiled conformation, and, hence, the formation of nanoparticles. Then, the formed nanoparticles are stabilized by cross-linking agents^[34-36]^. The most commonly employed cross-linking agent is glutaraldehyde. It was shown that the lowest required concentration of glutaraldehyde needed for the stabilization of nanoparticles is 40%, allowing the reaction to proceed for 24 h, ensuring the sufficient cross-linking of albumin amino groups^[35]^. Doxorubicin (DOX)-loaded HSA NPs were prepared by employing the desolvation method. Furthermore, HSA NPs retained their capacity for functionalization with targeting materials as well as active therapeutic agents [Figure 2]^[37]^.

### Thermal-induced aggregation

Thermal mediated unfolding of albumin can be induced by heating albumin solution, for instance at 65 °C. The procedure continues with protein-protein interactions. These interactions are hydrophobic, electrostatic, hydrogen bonding, and disulfide-sulfhydryl interchange interactions^[38-40]^. This method circumvents the potential toxicity, which may be attributed to the addition of organic solvents, in the desolvation method. Cyclopamine (CYC) and doxorubicin hydrochloride (DOX.HCL) were sequentially loaded in BSA NPs, implementing thermal-induced aggregation procedure. DOX.HCL, being water soluble, was mixed with BSA solution before the formation of NPs, while CYC was dissolved in ethanol and then added to the BSA solution. CYC is a hydrophobic drug, and hence the mechanism of CYC loading was shown to be a desolvation process. The mixture was magnetically stirred at 750 rpm in a 65 °C water bath. Then, the colloidal solution was ultra-filtered to remove any excess buffer or drug^[41]^.

### Self-assembly

The self-assembly of a protein into nanoparticles can be employed using several techniques. The mainstay in this technique is to increase the hydrophobicity of a water-soluble protein or to impart amphiphilic characteristics to the protein, through conjugation with a hydrophobic material^[42]^. In one study, HSA was activated and conjugated to sulfosuccinimidyl 4-(N-maleimidomethyl)-cyclo-hexane-1-carboxylate (sulfo-SMCC), and then it was conjugated to DOX.HCL. Furthermore, HSA was conjugated with octanal (C8) via reductive amination step, in the presence of sodium cyanoborohydride (NaCNBH_3_). The HSA-SMCC-DOX-C8 was self-assembled into HSA-DOX nanoparticles by adjusting the pH to 5.5, due to increased hydrophobicity by conjugated octanal^[43]^. Alternatively, the self-assembly process of albumin can be driven by protein unfolding induced by disulfide bond reduction, using disulfide bond reducing agents, for instance β-mercaptoethanol. Then, the addition of the hydrophobic drug allows the self-assembly of albumin into nanoparticles, due to non-covalent hydrophobic interactions arising between the drug and albumin^[44]^. Paclitaxel (PTX)-loaded HSA nanoparticles were successfully prepared by implementing the non-covalent protein-drug self-assembly method. First, PEGylated albumin was conjugated to a W peptide (Trp-Lys-Tyr-Met-Val-D-Met), for targeting of formyl peptide receptor overexpressed on triple negative breast cancer (TNBC). Then, glutathione (GSH), a disulfide bond reducing agent, was added to the modified HSA with elevating the temperature to 37 °C, to expose the hydrophobic binding sites of albumin. The formulation was first dialyzed to remove excess GSH, and then an ethanolic solution of PTX was added with the subsequent formation of NPs. This technique offers the advantage of producing redox-responsive nanocarriers, owing to the intermolecular disulfide bridges formed during the self-assembly process^[45]^.

### Albumin-bound technology

Albumin-bound technology (nab-technology) was introduced as an alternative technique for safe and effective systemic drug delivery formulation^[46,47]^. Cremophor-EL was used as a vehicle for the systematic delivery of paclitaxel, which induced anaphylactic reactions to various patients^[48]^. To overcome this limitation, paclitaxel was formulated bound to albumin by nab-technology. Initially, the drug is mixed with the aqueous solution of HSA, and then the mixture is passed through a high-pressure jet to form the drug-albumin nanoparticles^[49]^. These nanoparticles showed a size of 130 nm. The nab-paclitaxel was approved by the FDA, under the trade name of Abraxane®, for treatment of metastatic breast cancer. Similarly, for the treatment of drug-resistant pancreatic cancer, gemcitabine (GEM)-loaded albumin was fabricated using the same technique. First, to increase its hydrophobicity, GEM was coupled with myristoyl moiety, to produce GEM-C14. Then, the modified drug was dissolved in chloroform saturated with pure water. The resultant solution was further mixed with HSA solution. The mixture was homogenized at 2000 psi for nine cycles. Chloroform was removed by rotary evaporation under vacuum, syringe filtered and finally lyophilized to obtain powdered nanoparticles^[50]^.

## Albumin-based nanocarriers to overcome cancer drug resistance for various types of tumors

Albumin nanocarriers have been prepared, serving as drug delivery systems, to overcome resistance mechanisms developed by various tumors. In this regard, combined anticancer drug delivery, enhanced cellular uptake by gp60- and SPARC-mediated transcytosis, improved drug accumulation, and efficient delivery of labile therapeutics have been investigated [Table 1].

**Table 1:** Albumin-based nanoparticles employed to overcome cancer drug resistance

Drug	Particle size (nm)	PDI	Method	Type of cancer	Type of cancer drug resistance treated	Outcome	Ref.
Bovine serum albumin (BSA) based nanoparticles							
TCS and ABZ	98	0.18	Self-assembly	Lung cancer	Phosphorylation of caspase 9, upregulation of P-gp, and upregulation of α-tubulin	Inhibition of metastasis with an efficiency > 80% against A549/T tumor-bearing nude mice	[51]
CCM and DOX	131 (spherical)	N/A	High-pressure homogenization	Lung cancer	Poor drug uptake, accumulation, and decreased response to monotherapy	Enhanced cytotoxicity with around 80% cell death	[52]
QT and DTX	209	0.184	Antisolvent precipitation method	Breast cancer	Increased drug efflux by P-gp	4.27- and 1.87-fold reduction in the IC_50_ compared to free DTX and DTX-BSA-NPs, against MDA-MB-231 cells	[53]
CYC and DOX	151	0.206	Thermal induced aggregation	Breast cancer	Upregulation of P-gp	Enhanced accumulation of DOX, with only 2% survival of DOX-resistant MDA-MB-231 breast cancer cells	[41]
VE and PTX	106.9	0.172	Desolvation-ultrasonication method	Breast cancer	Increased drug efflux by P-gp	Enhanced cytotoxicity, as well as improve PTX tumor accumulation against MCF-7/ADR cells	[54]
Cur and DOX	99.94	0.193	Desolvation method	Breast cancer	Upregulation of P-gp	A lower cell viability was exhibited after the treatment with the Cur-DOX co-loaded albumin NPs, compared to the Cur loaded albumin NPs or the DOX loaded albumin NPs	[55]
Ce6 and DOX	HMSN: 231 BMHDC: 274	N/A	Biomineralization and Conjugation	Cervical cancer	Hypoxia-associated photodynamic therapy resistance	Reduction of Hela cell viability by more than 90%	[56]
disulfuram/copper complex and Rego	140.4	0.185	Hydrophobic drug induced co-assembly	Colorectal cancer	Poor drug accumulation and polarization of TAM to M2 phenotype	Downregulation of mannose receptors, and reduction in the population of M2 macrophages by up to 22%	[57]
DOX	60	0.23	Desolvation method	Uterine sarcoma	P-gp overexpression resulting in DOX resistance	DOX-DBSA-NPs, prepared by DMSO as desolvating agent, and DOX-SBSA-NPs, prepared by acetone, as the desolvating agent, exerted an enhanced cytotoxicity (IC_50_ = 0.39 and 0.25 μmol/L, respectively), with a lower IC_50_ than free DOX (IC_50_ = 2.09 μmol/L)	[58]
Human serum albumin (HSA) based nanoparticles							
DOX	496.4	0.213	Desolvation method	Neuroblastoma	ABCB1-mediated drug efflux	Enhanced DOX sensitivity and anticancer activity against vincristine adapted UKF-NB-3^r^VCR^1^ cells, but not DOX-resistant UKF-NB-3^r^DOX^2^0 cells, when treated with DOX-loaded albumin NPs	[59]
TRAIL and DOX	341.6	N/A	Self-assembly	Lung cancer	TRAIL or DOX monotherapy resistance	Enhanced apoptosis against H226 cells, compared to single drug-loaded nanoparticles	[43]
TRAIL and DOX	220	N/A	Desolvation method	Colon, breast, pancreatic cancer	TRAIL monotherapy resistance and drug efflux	99% cell-killing against CAPAN-1 cells	[37]
DTX	248.7	0.13	Albumin-coated nanocrystals	Ovarian cells	Poor drug uptake	Enhanced cell uptake by 2.5 folds after 1 h and around 8 folds in 3 h, via SPARC-mediated mechanism	[60]
DTX and IR-780	146.5	N/A	Self-assembly	Prostate cancer	Poor efficacy of PTT and PDT monotherapy	Increased temperature up to 47.5 °C, with an irreversible tumor damage	[61]
Human survivin-specific miRNA plasmid	220	0.04	Desolvation method	Colorectal cancer	Overexpression of Survivin	50% reduction in survivin expression, initiation of apoptosis, and reduction of cell viability by up to 60% at 2 Gy, with combined radiotherapy	[33]
PTX	118.8	0.221	Hydrophobic drug induced co-assembly	TNBC	Lack of the expression of HER2, estrogen and progesterone receptors	Enhanced cellular uptake, and a lower IC_50_ (553.5 ng/mL) compared to free PTX (3612.1 ng/mL)	[45]
Cat, Ce6 and PTX	100	N/A	Hydrophobic drug induced co-assembly	Breast cancer	Poor intra-tumoral penetration, tumor hypoxia	Enhanced PDT, due to generation of oxygen in situ by the action of catalase	[62]
GEM	150	N/A	Albumin-bound technology	Pancreatic cancer	low hENT1 expression; Poor GEM uptake	Reduced tumor volume and weight compared to free GEM	[50,63]

TCS: trichosanthin; ABZ: albendazole; CCM: curcumin; DOX: doxorubicin; QT: quercetin; DTX: docetaxel; CYC: cyclopamine; PTX: paclitaxel; VE: vitamin E; Rego: regorafenib; Cur: curcumin; DOX-DBSA-NPs: doxorubicin loaded doughnut shaped bovine serum albumin nanoparticles; DOX-SBSA-NPs: doxorubicin loaded spherical shaped bovine serum albumin nanoparticles; TRAIL: tumor necrosis factor (TNF)-related apoptosis-inducing ligand; CAPAN-1: human pancreatic ductal adenocarcinoma cell line; SPARC: secreted protein acidic and rich in cysteine; TNBC: triple negative breast cancer; PDT: photodynamic therapy; PTT: photothermal therapy; HER2: human epidermal growth factor receptor 2; Cat: catalase; hENT1: human equilibrative nucleoside transporter 1; GEM: Gemcitabine

### Breast cancer

Drug efflux is a major pathway for the development of multi-drug resistant breast cancer. The primary mechanism has been found to be the upregulation of the ATP-binding cassette (ABC), which possesses the capacity to pump chemotherapeutic drugs out of the cancer cells^[64]^. A combination treatment with an efflux pump inhibitor would be a promising candidate^[13,65]^. Cyclopamine (CYC), a hedgehog signaling pathway inhibitor, was found to regulate the expression of ABCB1, which is also known as P-glycoprotein (P-gp)^[66]^. CYC was effectively combined with doxorubicin (DOX) for reversal of drug resistance in breast cancer^[67]^. DOX and CYC were successfully co-loaded in BSA nanoparticles (Drug loading 1.2% and 9.1%, respectively) using a thermal induced self-assembly process. To its advantage, BSA NPs internalized in tumor cells via gp60- and SPARC-mediated pathways. Notably, BSA-CYC-DOX NPs showed decreased levels of P-gp, as well as enhanced accumulation of DOX, which were consistent with only 2% survival of DOX-resistant MDA-MB-231 breast cancer cells^[41]^. Similarly, Quercetin (QT), a P-gp inhibitor, was co-loaded with Docetaxel (DTX) in BSA NPs, resulting in 4.27- and 1.87-fold reduction in the IC_50_ compared to free DTX and DTX-BSA-NPs, against MDA-MB-231 cells. Moreover, these nanoparticles showed the highest fluorescence intensity inside tumor cells, attributed to the effective inhibition of P-gp. DTX-QT-BSA-NPs were prepared using anti-solvent precipitation method, with an entrapment efficiency of 75.18% and 68.09% for DTX and QT, respectively^[53]^.

Another resistance mechanism towards photodynamic therapy (PDT) was found to be the hypoxic nature of the tumor microenvironment (TME)^[68-74]^. To overcome this hypoxic TME, catalase was used to produce oxygen in-situ, through the specific decomposition of H_2_O_2_ to O_2_^[75]^. However, the systematic administration of catalase was limited by the action of blood circulating proteases^[76-80]^. Therefore, catalase was co-loaded with paclitaxel (PTX) using pre-modified HSA-Ce6, to form HSA-Ce6-Cat-PTX nanoparticles. The nanoparticles, which were prepared through the self-assembly procedure of HSA, were formed by the addition of the hydrophobic drug, PTX. The produced nanoparticles (~100 nm) showed an enhanced PDT, owing to the effective O_2_ by catalase. Moreover, the catalase maintained about 70% of its activity after 24 h incubation of the nanoparticles with protease K^[62]^.

Triple negative breast cancer (TNBC) lacks the expression of human epidermal growth factor receptor 2 and estrogen and progesterone receptors, which may compromise therapeutic treatment^[45]^. Active targeting was exploited to deliver PTX to TNBC. TNBC overexpresses formyl peptide receptor^[81,82]^, which could be targeted with a W peptide (Trp-Lys-Tyr-Met-Val-D-Met)^[83,84]^. A disulfide bond reduction method^[85,86]^ was employed to induce the self-assembly of Wpep-HSA, following the addition of the hydrophobic drug, PTX. Enhanced intracellular release of Wpep-HSA-PTX NPs was observed, due to redox-responsive behavior, which was attributed to elevated levels of endogenous GSH^[87]^. The stimuli-responsive drug release pattern was linked to the formation of intermolecular disulfide bonds between HSA NPs^[45]^. Furthermore, these nanoparticles showed an enhanced cellular uptake, and exhibited a lower IC_50_ (553.5 ng/mL) compared to free PTX (3612.1 ng/mL). The favorable anti-proliferative effects, stimuli-responsive drug release, and enhanced pharmacokinetic profile of the Wpep-HSA-PTX NPs demonstrated an effective strategy to overcome inadequacies in TNBC treatment. In another investigation, DOX-resistant MCF-7/ADR cells did not respond to TRAIL-HSA NPs therapy. However, the resistant cells were found to respond to the treatment of transferrin-conjugated TRAIL/DOX HSA-NPs. MCF-7/ADR cells possess an efficient efflux mechanism. Therefore, active targeting, to ensure drug internalization and subsequent accumulation, was sought. Transferrin receptors are overexpressed on the MCF-7 cells, and, thus, it was shown that multi-drug resistance (MDR) could be overcome through delivery of TRAIL and DOX, through transferrin-receptor mediated endocytosis^[37]^.

### Lung cancer

**Figure 3:**
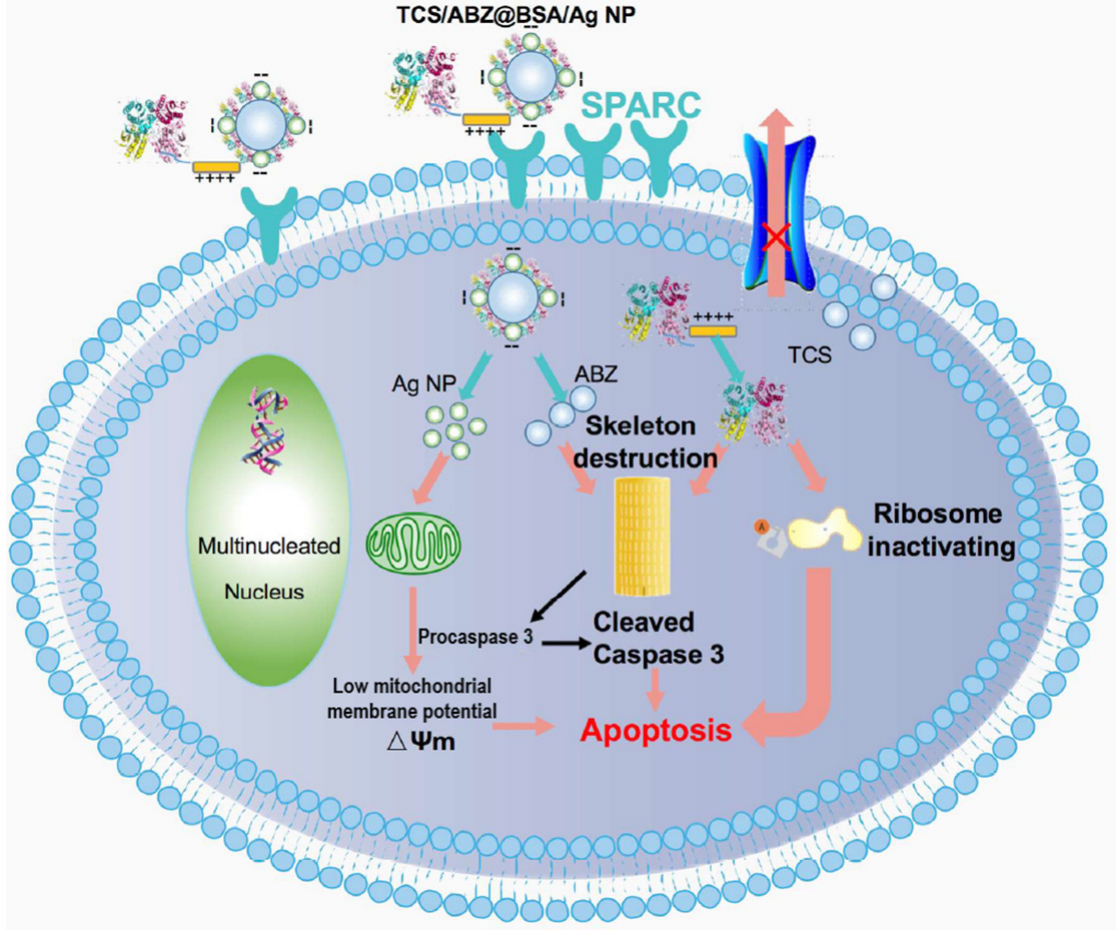
Schematic representation of TCS/ABZ-BSA/Ag NPs internalization utilizing SPARC-mediated endocytosis, as well as antitumor mechanisms against A549/T cells^[51]^

Phosphorylation of caspase 9^[88]^, upregulation of α-tubulin^[89]^, and upregulation of P-gp^[90,91]^ are all resistance mechanisms encountered with metastatic lung cancer. Trichosanthin (TCS) is a ribosome-inactivating protein^[92,93]^, which re-sensitizes MDR cancer cells by the dephosphorylation of caspase 9. It was shown that TCS could reverse resistance in MDR cancer cells in combination with paclitaxel^[88]^. Moreover, TCS has the potential of preventing the polymerization of tubulins. In an attempt to synergistically overcome MDR lung cancer, albumin-coated silver nanoparticles were synthesized for the co-delivery of albendazole and the recombinant fusion (rTL) protein of trichosanthin with low molecular weight protamine (LWMP). The cationic rTL was self-assembled on the negatively charged albendazole-loaded BSA/Ag NPs via electrostatic interactions. Exploiting SPARC-mediated cellular internalization, these nanoparticles resulted in dephosphorylation of caspase 9, downregulation of P-gp, and downregulation of α-tubulin. Additionally, the mitochondrial membrane potential was reverted to low voltage-mitochondrion, indicating the collapse of the mitochondrial membrane and subsequent initiation of apoptosis [Figure 3]^[94]^. Finally, TCS-containing nanoparticles inhibited metastasis with an efficiency > 80% against A549/T tumor-bearing nude mice, confirming the efficiency of TCS as a treatment in resistant and metastatic cancers^[51]^.

Tumor necrosis factor (TNF)-related apoptosis-inducing ligand (TRAIL), which binds to the overexpressed death receptor 4 and 5, exhibited limited efficacy as a monotherapy in resistant cancer cells^[95-98]^. However, when combined with chemotherapeutic drugs, TRAIL was found to be an effective treatment^[99-101]^. To overcome resistance to TRAIL, DOX and TRAIL were combined to fabricate a single nanomedicine-based system. DOX was conjugated to HSA and self-assembled by virtue of increased hydrophobicity, secondary to conjugation of octanoic acid to HSA. TRAIL was then added to coat the DOX-loaded HSA NPs through sonication in an ice bath. The inhalable nanoparticles were retained in the lung for three days, exerting synergistic apoptotic activity against H226 cells, compared to DOX-loaded HSA NPs or TRAIL-loaded HSA nanoparticles^[43]^. An injectable formulation of Curcumin (CCM) and DOX-co-loaded BSA NPs was fabricated by high pressure homogenization/nab-technology for the treatment of metastatic lung cancer. The synergistic combination (Combination index: 0.6069) showed an enhanced cytotoxicity with around 80% cell death. These nanoparticles exploited the ability of BSA to target gp60, which allowed their internalization into the cancer cells^[52]^.

### Pancreatic cancer

Pancreatic ductal adenocarcinoma (PDAC) is an aggressive type of cancer. The first line agent for treatment of PDAC is gemcitabine (GEM)^[102]^. However, GEM, a hydrophilic nucleoside analog, utilizes human nucleoside transporters (hNT) for drug transport into cancer cells^[103]^. One main factor for PDAC chemoresistance is the low expression of one of hNTs, namely the human equilibrative nucleoside transporter (hENT)^[104,105]^. A promising strategy was investigated to increase the lipophilicity of GEM, conjugating a myristoyl moiety (C14) with the 4-amino group of GEM (GEM-C14). GEM-C14 was loaded in HSA NPs produced by albumin-bound technology (nab-technology)^[50]^. Thus, GEM-HSA NPs would overcome the low expression of hENT, utilizing the active targeting properties of HSA (gp60 and SPARC targeting) receptor mediated endocytosis. GEM-HSA NPs showed marked tumor growth inhibition (more reduced tumor volume and weight) compared to free GEM and control group^[63]^.

As previously demonstrated, TRAIL is an effective treatment only in combination with other anticancer agents, for instance the chemotherapeutic agent DOX. DOX-loaded HSA NPs, prepared via desolvation method and cross-linked with glutaraldehyde, were surface-decorated with TRAIL and transferrin, by virtue of the amine groups of HSA NPs. Transferrin allowed the NPs to be actively internalized in CAPAN-1 (pancreatic cell line model, less sensitive to necrosis and apoptosis), via receptor mediated endocytosis. Furthermore, the combination of TRAIL and DOX exerted more apoptotic activity (> 80% cell-killing), especially due to the action of TRAIL, at lower doses of TRAIL and DOX, *in vivo* compared to TRAIL monotherapy. However, on administration of TRAIL/Tf/DOX HSA NPs, 99% CAPAN-1 cell-killing was demonstrated by FACS analysis^[37]^.

Immune evasion is one of the resistance mechanisms developed by various tumors^[106]^. One way to avoid immune recognition by cancer cells is the polarization of tumor-associated macrophages (TAM) from the M1 phenotype to the immunosuppressive, M2 phenotype. To overcome this mechanism of resistance in PDAC, a nano-formulation of paclitaxel-loaded albumin was prepared by albumin-bound technology (nab-paclitaxel). Targeting gp60 and SPARC, the internalization of nab-paclitaxel into TAM induced the activation of M1 Phenotypes^[107]^.

### Colorectal cancer

Resistance of colorectal cancer cells against radiotherapy was assumed to be mediated by some molecular targets. One example is survivin, which is an inhibitor of apoptosis^[108]^. Knockdown of survivin via plasmid-mediated RNA interference is a promising strategy^[109,110]^. However, plasmids may be unstable, due to degradation by nucleases. To overcome this challenge, it was found that the loading of a plasmid in HSA NPs, prepared by a desolvation technique and stabilized via cross-linking with glutaraldehyde, is a successful approach. The plasmid loaded in HSA NPs was stable against DNase 1, compared to free plasmid. Moreover, at a concentration corresponding to 300 µg of the plasmid, 50% downregulation of survivin expression was observed. The reduced expression of survivin resulted in the initiation of apoptosis with reduced cell viability, which was further enhanced with combined radiotherapy for up to 60% reduced cell viability at 2 Gy. A greater reduction in cell viability was observed with increasing doses of radiotherapy (at 8 Gy)^[33]^.

The tumor microenvironment may be associated with chemoresistance, as well as tumor progression and angiogenesis. It was found that the polarization of tumor-associated macrophages (TAM) to the M2 phenotype may induce chemotherapeutic drug resistance^[111]^. Interestingly, mannose receptors (MR) were found to be overexpressed on M2 macrophages, which can be targeted with mannose-conjugated nanoparticles, and subsequent internalization of the nanocarriers into the cell via receptor mediated endocytosis^[112-115]^. In this regard, mannose- functionalized BSA (Man-BSA) NPs were synthesized, co-encapsulating disulfuram/copper complex (DSF/Cu), as well as regorafenib (Rego). The nanoparticles were prepared by hydrophobic mediated self-assembly, induced by the addition of the hydrophobic drugs, secondary to NabH_4_/urea protein-unfolding. Man-BSA NPs exhibited three-fold higher cellular uptake than BSA NPs, due to the overexpression of MR receptors on the drug-resistant colon cancer cell line: HCT8/ADR cells. The combinatorial effect of the drug-loaded BSA NPs resulted in an effective therapeutic outcome through a number of pathways: (1) elevated reactive oxygen species (ROS) generation; (2) anti-angiogenic activity, mediated by regorafenib; (3) induction of autophagy and apoptosis pathways; and, more importantly; and (4) the reversion of M2 macrophages to M1 phenotype, which was mainly by regorafenib and further enhanced by the dual drug-loaded BSA NPs and was evident by downregulation of mannose receptors. Moreover, the Man-BSA NPs showed the strongest reduction in the population of M2 macrophages, up to 22%. These results highlight the significance of targeting the tumor microenvironment, which can enhance the treatment outcomes in MDR cancers^[57]^.

### Prostate cancer

Castration-resistant prostate cancer is presented with continuous rise in the serum levels of the prostate-specific antigen (PSA) or development of new metastases^[116]^. Combined treatment with photothermal and photodynamic therapy plus chemotherapy offers advantages over monotherapy^[117,118]^. However, limited by poor drug pharmacokinetics and reduced tumor accumulation, the clinical application of these agents is severely compromised. In an attempt to overcome drug resistance and improve the therapeutic outcome of these agents, a nanomedicine-based system was developed to encapsulate both docetaxel (DTX) and a near-infrared dye, IR-780. The hydrophobic drugs triggered the self-assembly of HSA into nanoparticles, following β-mercaptoethanol-mediated unfolding of HSA. The dual drug loaded HSA NPs were taken up by tumor cells and exhibited an increased temperature up to 47.5 °C, which was enough for irreversible tumor damage. Additionally, these nanoparticles allowed an efficient generation of ROS. Subsequently, HSA-IR780-DTX NPs with NIR laser irradiation showed marked reduction in cell viability at 1 and 1.5 μg/mL concentrations, compared to monotherapy, which was mainly attributed to IR-780^[61]^.

### Cervical cancer

To overcome tumor hypoxia and enhance photodynamic (PDT) therapy in cervical carcinoma, DOX- and Ce6-loaded mesoporous silica nanoparticles were synthesized and further conjugated to BSA-MnO_2_ NPs through a disulfide linkage to produce BSA-MnO2-HMSNs-DOX-Ce6 (BMHDC) nanoparticles. BSA-MnO_2_ NPs were prepared by a biomineralization strategy^[119]^. These gated nanoparticles showed a stimuli-responsive behavior and an on-demand cargo release. BSA-MnO_2_ acted not only to control drug release in response to pH (5.5 and 7.4) and GSH of the TME, but also generated oxygen utilizing endogenous H_2_O_2_, relieving tumor hypoxia to enhance PDT treatment. Moreover, intracellular delivery of BMHDC nanoparticles resulted in a 2.1-fold increase in intracellular DOX, compared to free DOX, as well as enhanced oxygen production. Interestingly, only 62% of the Hela and L929 cells died when treated with HMSNs-DOX-Ce6 only. Cell viability was further reduced, where more than 90% cells died, when the cells were treated with BMHDC nanoparticles and irradiated with NIR laser, suggesting the role of BSA-MnO_2_ in enhancing PDT treatment^[56]^.

### Ovarian cancer

Docetaxel (DTX) is an alternative treatment for ovarian cancer with fewer side effects than paclitaxel (PTX). However, drug-resistance can develop as a consequence of drug efflux, and thereby reduce drug accumulation at the tumor site. Albumin is exploited to facilitate the intracellular delivery of DTX to P-gp expressing NCI/ADR-RES cells. Initially, DTX was formulated as nanocrystals stabilized with Pluronic F127, then incubated with HSA water solution, to produce the DTX-loaded HSA NPs. The DTX-HSA NPs were more taken up (2.5 folds after 1 h and ~8 folds in 3 h) by the cancer cells via SPARC-mediated mechanism, bypassing drug efflux by P-gp. This resulted in an improved cytotoxic effect of DTX-HSA NPs over 4 h period compared to free DTX, showing extended release behavior intracellularly^[60]^.

### Uterine sarcoma

Uterine sarcoma is a type of cancer which develops at the muscular sites of the uterus. MES-SA/DX-5 is a 100-fold DOX-resistant cell line model of uterine sarcoma, which overexpresses P-gp^[120]^. DOX-loaded BSA NPs were prepared via desolvation technique. DMSO was employed to produce doughnut-shaped NPs (DBSA-NPs) and compared to spherical-shaped nanoparticles (SBSA-NPs). No difference was observed between MDR cell line and the non-resistant cell line, MOLT-4. Moreover, the cytotoxicity of both formulations, DOX-DBSA-NPs and DOX-SBSA-NPs, showed comparable results (IC_50_= 0.39 and 0.25 μmol/L, respectively), which were lower than free DOX (IC_50_ = 2.09 μmol/L), indicating inhibition of the proliferation of MDR cells and the bypassing of P-gp drug efflux^[58]^.

## Future perspectives and challenges

Nanotechnology-based drug delivery is a promising strategy to overcome cancer drug resistance^[14]^. Albumin nanocarriers are attracting considerable attention, provided by their favorable characteristics compared to other nanomaterials. However, some drawbacks may hinder their application. For instance, the application of bovine serum albumin may result in immunogenic reactions, being from animal source^[121]^. Moreover, organic solvents, which are implemented in the fabrication of albumin nanoparticles, may compromise their safety. For example, the toxicity profiles of glutaraldehyde^[122]^, employed for cross-linking of albumin nanoparticles, or β-mercaptoethanol^[123]^, used to induce disulfide bond reduction for subsequent self-assembly, may hinder the application of the produced nano-formulations^[44]^. On the other hand, the stability of albumin may be compromised by some nano-fabrication conditions. For instance, it was shown that the stability of albumin is compromised on the application of high-pressure homogenization technique (nab-technology)^[124]^.

**Figure 4:**
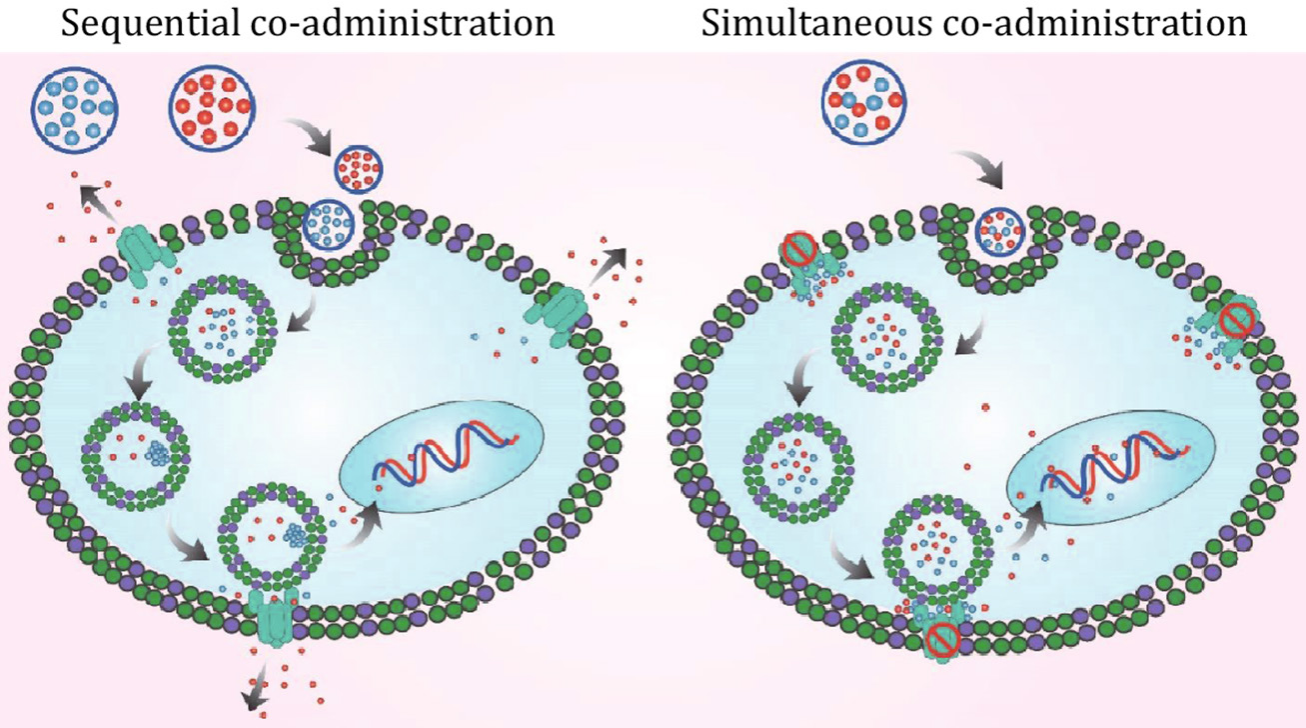
A schematic presentation showing the effective inhibition drug efflux inhibition by tuned drug administration and release mechanisms, mediated by Cur and DOX co-loaded albumin NPs^[55]^. Cur: curcumin; DOX: Doxorubicin; NPs: nanoparticles

Tuned drug release could be a potential strategy to overcome cancer drug resistance. The single loaded curcumin (Cur) albumin NPs and single DOX albumin NPs were administered sequentially, as well as simultaneously, to the MCF-7 resistant breast cancer cells. Although the simultaneous administration of both albumin NPs exhibited an increased accumulation of DOX and an enhanced cytotoxicity compared to the sequential administration, a lower Cur accumulation was demonstrated. This led to the limited P-gp inhibition by Cur. The challenging aspect was the internalization and release manner of Cur in lysosomes. The low pH exhibited by the lysosome caused Cur to aggregate, a phenomenon known as lysosomotropism^[125]^, which compromised the efficacy of Cur. On the contrary, both DOX and Cur co-loaded albumin NPs, showing a concomitant drug release, displayed a more efficient cell killing. Interestingly, the release of DOX exhibited a buffering capacity, elevating the lysosomal pH, which caused lysosomal drug release and kept the Cur in a dispersed form. Once both were released in the cytosol, Cur, in its dispersed form, efficiently inhibited P-gp, which prevented the efflux of DOX from the cancer cells [Figure 4]^[55]^.

Despite the significance of ABCB1 transporter, as cancer drug resistance target, it was shown that ABCB1 inhibitors, as well as DOX-loaded albumin nanocarriers, failed to achieve therapeutic goal *in vitro* against drug-resistant neuroblastoma cells. It was shown that the DOX-resistant UKF-NB-3^r^DOX^20^ cells were not re-sensitized by either an ABCB1 inhibitor, zosuquidar, or the DOX-loaded albumin nanoparticles. On the contrary, vincristine adapted UKF-NB-3^r^VCR^1^ cells, which overexpress ABCB1 transporter, were sensitive to the DOX-loaded albumin nanoparticles. It was conferred that DOX-resistant UKF-NB-3^r^DOX^20^ cells possess multiple resistance mechanisms, which were not sufficiently targeted with an efflux transporter inhibitor or albumin nanocarriers. The internalization of albumin nanoparticles, which was efficient in the treatment of the UKF-NB-3^r^VCR^1^ cells, is mediated by binding of albumin to the albumin receptors overexpressed on tumor cells. However, variations in drug loading mechanisms as well as the kinetics of drug release may influence the therapeutic efficiency^[126,127]^. The findings of this study provided primary evidence for the importance of understanding personalized therapy against drug-resistant cancer, provided by more comprehensive insights about cancer drug resistance mechanisms^[59]^. For instance, elucidating the mechanism of resistance by certain biomarkers may enhance albumin-based drug delivery, based on the specific characteristics of the tumor. Moreover, targeting multiple cancer drug resistance mechanisms, through combined drug use, may represent an alternative strategy. In addition, active targeting, by exploiting the functionalization properties of albumin, may pave the way for more efficient drug delivery for specific tumors, hence overcoming drug resistance. Nano-theranostics, based on albumin nanoparticles, are a promising approach that allow personalized treatment utilizing imaging and therapeutic modalities. Finally, more studies are required on other drug-resistant tumors, investigating the possibility of targeting multiple resistance mechanisms, tumor imaging, and, more importantly, site-specific drug delivery.

## Conclusion

Albumin nanocarriers have shown advantageous characteristics, including biodegradability, biocompatibility, and favorable toxicological profiles. Albumin was able to target overexpressed gp-60 and SPARC receptors, allowing an enhanced drug uptake and bypassing drug efflux mechanisms. Moreover, albumin nanocarriers improved the stability of sensitive therapeutic cargos, such as nucleic acids. Albumin nanocarriers demonstrated the capacity for functionalization with targeting materials and active therapeutics. Furthermore, stimuli-responsive drug release can be implemented utilizing albumin nanoparticles. Collectively, nanomedicine-based albumin drug delivery is a promising strategy to overcome cancer drug resistance.

## Declarations

### Copyright

© The Author(s) 2020.
